# Association Study: The Aminopeptidase a Gene and Essential Hypertension

**Published:** 2005-06

**Authors:** Morihiko Sano, Nobuhiro Kuroi, Tomohiro Nakayama, Naoyuki Sato, Yoichi Izumi, Masayoshi Soma

**Affiliations:** 1 *Nihon University School of Medicine, Japan;*; 2 *Division of Receptor Biology, Advanced Medical Research Center, Japan;*; 3 *Second Department of Internal Medicine Nihon University School of Medicine, Tokyo, Japan*

**Keywords:** essential hypertension, aminopeptidase A, single nucleotide polymorphism, haplotype, genetic, association study

## Abstract

Aminopeptidase A (APA) cleaves the N-terminal aspartyl acid residue of angiotensin II (Ang II) to produce angiotensin III (Ang III). It has been reported that the APA knockout mouse exhibits elevated blood pressure. Therefore, the APA gene is thought to be a susceptibility gene for essential hypertension (EH). However, extensive studies have yet to define the relationship between the APA gene and EH. The aims of this study were to genotype some of the single nucleotide polymorphisms (SNPs) for the human APA gene and to perform a haplotype-based case-control study to further assess the association between and the APA gene and EH. We performed a genetic association study using SNPs in 227 EH patients and 221 age-matched normotensive (NT) individuals. Although the overall distribution of the genotype did not significantly differ between the EH and NT groups when the entire group of subjects were evaluated, the frequency of rs2290105 did differ between the two when just women were included in the analysis. The haplotype-based case-control analysis also revealed a significant difference between the women of the EH and NT groups. The A-T-A-C haplotype was significantly higher in the EH versus the NT group. These results suggest that rs2290105 and the A-T-A-C haplotype of the APA gene are genetic markers for EH, and that APA or a neighboring gene might be a susceptibility gene for EH.

## INTRODUCTION

Angiotensinogen is cleaved by renin to generate angiotensin I (Ang I), which is further converted to angiotensin II (Ang II) by the angiotensin converting enzyme (ACE). Ang II is the fundamental peptide of the renin-angiotensin system (RAS), which induces constriction of blood vessels and increases the sodium and water retention, thus leading to an increase in blood pressure. Previous studies using mice that had a disruption of the genes responsible for the generation of angiotensin and the encoding of Ang II have provided information on the function of these genes. Aminopeptidase A (APA) cleaves the N-terminal aspartyl acid residue of Ang II to produce angiotensin III (Ang III). APA is the so-called angiotensinase and several studies have reported that purified APA functions as a hypotensive factor and that inhibitors of APA are hypertensive factors *in vivo*. A study that examined APA deficient mice found that the baseline values for systolic blood pressure (SBP) and mean blood pressure (MBP) were higher in these animals. The blood pressure level for the mice with the targeted homozygous APA gene (APA-/-) was found to be significantly higher than that seen in the heterozygous (APA+/-) and wild types (APA+/+). These results suggest that APA is involved in blood pressure regulation, and that the metabolism of Ang II is a very important factor in the physiological regulation of blood pressure (1-5). Although the APA gene is thought to be a susceptibility gene for hypertension, there have been no studies examining that examined the association between the APA gene and essential hypertension (EH).

The APA gene is also listed as the negative primary endpoint (ENPEP) gene in the NCBI online database. The human APA gene is located on chromosome 4q25. This gene contains 18 exons, is interrupted by 17 introns, and has several single nucleotide polymorphisms (SNPs) (6, 7). High blood pressure or hypertension affects 25% of most adult populations and is an important risk factor for death from stroke, myocardial infarction and congestive heart failure. The majority of hypertensive cases are classified as primary and are referred to as essential hypertension (EH). EH is thought to be a multifactorial disease (8). However, there are a few reports that identify the susceptibility genes of EH as angiotensinogen (9), or angiotensin converting enzyme (10, 11).

The aims of this study were to genotype some of the single nucleotide polymorphisms (SNPs) for the human APA gene and to perform a haplotype-based case-control study to further assess the association between and the APA gene and EH.

## METHODS

### Subjects

This study included a group of 227 patients that were diagnosed with EH. A positive diagnosis required the patient to have a seated SBP above 160 mmHg and/or diastolic blood pressure (DBP) above 100 mmHg on 3 occasions within 2 months after their first medical examination. None of the patients were using anti-hypertensive medication and any of the subjects diagnosed with secondary hypertension were excluded. We also included 221 normotensive (NT) healthy individuals as controls. None of the NT participants had a family history of hypertension, and they all had SBP and DBP below 130 and 85 mmHg, respectively. A family history of hypertension was defined as prior diagnosis of hypertension in grandparents, uncles, aunts, parents or siblings. Both groups were recruited from the northern area of Tokyo, and informed consent was obtained from each individual as per the protocol approved by the Human Studies Committee of Nihon University (12).

### Biochemical analysis

The methodology of the Clinical Laboratory Department of Nihon University Hospital was used to measure all plasma total cholesterol and HDL-cholesterol concentrations, and serum creatinine and uric acid concentrations (13).

### Genotyping

Based on the allelic frequency data for the registered SNPs from the National Center for Biotechnology Information (NCBI) website and from Applied Biosystems-Celera Discovery System, we chose SNPs with minor allele frequencies of 20% or greater. This criterion was chosen since SNPs with a high frequency of the minor allele are very useful as genetic markers in genetic association studies.

**Figure 1:**
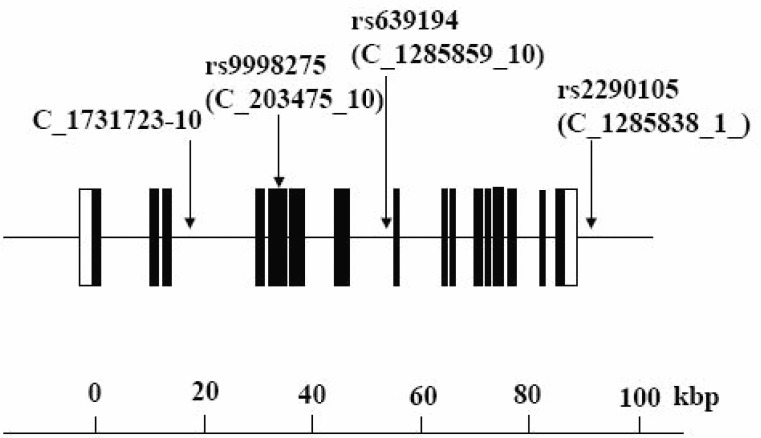
Organization of the human APA gene and location of the SNPs used for the association study. Closed black boxes indicate exons (coding region), closed white box indicate exons (non coding region) and lines indicate introns.

We selected 4 SNPs in the introns of the human APA gene as markers for the genetic association experiment (Fig. 1). The minor allele frequencies for each of the SNPs among the Japanese subjects were >10% (screening estimate from the Celera Company), which indicates that they all should be effective genetic markers. All SNPs were confirmed using the dbSNP on the NCBI website and the Applied Biosystems-Celera Discovery System. The accession numbers were as follows: C_1731723_10 (no registration in the NCBI), rs9998275 (C_203475_10), rs639194 (C_1285859_10), and rs2290105 (C_1285838_1) (Fig. 1). Genotypes were determined using Assays-on-Demand kits (Applied Biosystems, Branchburg, NJ) together with TaqMan R PCR. When allele-specific fluorogenic probes hybridize to the template during the polymerase chain reaction (PCR), the 5’ nuclease activity of the Taq polymerase can discriminate alleles. Cleavage results in increased emission of a reporter dye that otherwise is quenched by the dye TAMRA. Each 5’ nuclease assay requires two unlabeled PCR primers and two allele-specific probes. Each probe is labeled with a reporter dye (VIC and FAM) at the 5’ end and TAMRA at the 3’ end. Amplification by PCR was carried out using TaqMan Universal Master Mix (PE Biosystems) in a 25 u1 reaction volume with final total concentrations of 50 ng DNA, 700 nM primer, and 100 nM probe. Thermal cycling conditions consisted of 95°C for 10 min, and then 40 cycles of 92°C for 15 sec and 60°C for 1 min in a GeneAmp 9700 system.

All 96-well plates contained 80 samples of unknown genotype, six known allele 1 homozygotes, six known allele 2 homozygotes, and four reactions with reagents but no DNA. The homozygote and control samples without DNA were required for the SDS 7700 signal processing that is outlined in the TaqMan Allelic Discrimination Guide (PE Biosystems). Direct sequencing, single-stand conformation polymorphism (SSCP), or denaturing high pressure liquid chromatography were used to confirm control sample genotypes. PCR plates were read on the ABI 7700 instrument in the end-point analysis mode of the SDS version 1.6.3 software package (ABI). Genotypes were visually determined by comparison with the dye-component fluorescent emission data shown in the X-Y scatter-plot of the SDS software. Genotypes were also automatically determined by the signal processing algorithms in the software. Results of both scoring methods were saved to two output files for later comparison.

### Haplotype-based case-control study

We performed haplotype analysis on the 4 SNPs. Based on the genotype data of the 4 genetic variations, the frequency of each haplotype was estimated using the expectation/maximization (EM) algorithm (14, 15). For the haplotype-based case-control study determinations, SNPAlyze version 3.2 was used (Dynacom Co., Ltd., Yokohama, Japan), which is available from their website at http://www.dynacom.co.jp/products/package/snpalyze/index.html.

### Statistical analysis

Data are shown as mean ± SD. Differences between the EH and NT groups were assessed by analysis of variance (ANOVA) followed by a Fisher’s protected least significant difference (PLSD) test. Hardy-Weinberg equilibrium was assessed by a chi-square analysis. When the sizes of the expected values were small (below 2.0), the genotypes were combined (16). The overall distribution of the SNP alleles was analyzed by 2 × 2 contingency tables, and the distribution of the SNP genotypes between the EH patients and NT controls was tested using a 2-sided Fisher exact test and multiple logistic regression analysis. Statistical significance was established at *p*<0.05. The threshold value of the frequencies of the haplotypes included in the analysis was set to 1/2n (n: numbers of subjects in each group), as suggested by Excoffier and Slatkin (17). All haplotypes below the threshold value were excluded from the analysis. Overall distribution of haplotypes was analyzed using 2× m contingency tables with a value of *p*<0.05 considered to indicate statistical significance. The p value significance of each haplotype was determined by the chi-square analysis and permutation method using the software SNPAlyze version 3.2 (16).

## RESULTS

Table 1 shows the clinical features for the EH patients and the NT controls. The SBP and DBP were significantly higher in the EH group as compared to that seen in the NT group. Age, body mass index (BMI), pulse rate, serum concentrations of creatinine and uric acid, and plasma concentrations of total cholesterol did not significantly differ between the two groups.

**Table 1:** Characteristics of study participants

	Total			Male			Femals		
	NT	EH	*P* Value	NT	EH	*P* Value	NT	EH	*P* Value

Number of subjects	221	227		143	143		78	84	
Age (years)	51.1 ± 8.4	51.0 ± 5.5	0.924	51.7 ± 6.1	50.8 ± 5.6	0.185	49.9 ± 11.5	51.4 ± 5.3	0.288
BMI (kg/m^3^)	23.4 ± 2.9	23.7 ± 2.6	0.240	23.6 ± 2.8	23.8 ± 2.4	0.461	23.0 ± 3.1	23.5 ± 2.9	0.297
SBP (mmHg)	112.6 ± 10.7	172.6 ± 20.1	<0.001	113.1 ± 10.1	170.9 ± 19.4	<0.001	111.6 ± 11.7	175.6 ± 21.0	<0.01
DBP (mmHg)	69.7 ± 8.7	106.2 ± 12.8	<0.001	70.5 ± 8.1	107.5 ± 12.6	<0.001	68.1 ± 9.7	103.8 ± 13.0	<0.001
Pulse (beats/mm)	73.9 ± 12.3	75.2 ± 13.1	0.351	74.1 ± 13.6	74.8 ± 13.8	0.697	73.6 ± 9.4	75.9 ± 12.0	0.253
Creatinine (mg/dl)	0.8 ± 0.2	0.9 ± 0.2	0.228	0.9 ± 0.2	0.9 ± 0.2	0.173	0.7 ± 0.1	0.7 ± 0.2	0.536
Total cholesterol (mg/dl)	203 ± 43.9	209.3 ± 39.0	0.179	198.3 ± 42.0	202.5 ± 37.0	0.390	213.8 ± 45.7	220.2 ± 39.9	0.349
HDL cholesterol (mg/dl)	56.0 ± 17.1	58.0 ± 17.7	0.257	53.4 ± 15.2	53.8 ± 16.3	0.867	60.4 ± 19.3	64.7 ± 17.9	0.167
Uric acid (mg/dl)	5.8 ± 4.7	5.6 ± 1.6	0.488	6.0 ± 1.4	6.1 ± 1.5	0.450	5.5 ± 7.7	4.7 ± 0.14	0.335
Alcohol consumption (%)	60.8	67.8	0.126	72.8	81.6	0.066	33.9	38.6	0.569
Smoking (%)	40.9	54.5	0.004	49.2	64.2	0.008	22.8	33.7	0.159

BMI, Body mass indes; SBP, Systolic blood pressure; DBP, Diastolic blood pressure; HDL, High density lipoprotein; NT, Normotension; EH, Essential hypertension.

We performed an association study using 4 SNPs. Table 2 shows the genotype distributions of the 4 SNP variants and allelic frequencies. The overall distribution of the genotypes did not significantly differ between the EH and NT groups. However, there was a difference noted for the allelic frequency of the SNP rs2290105 between women in the EH and NT groups (*p* value=0.0409), with the allelic frequency of the minor allele in the EH group higher than that seen for the NT group.

**Table 2:** Genotype Distribution in Normotensives (NT) and Patients with Essential hypertension (EH)

		Total			Male			Female		
		NT	EH	*P Value*	NT	EH	*P Value*	NT	EH	*P Value*

Number of participants		221	227		143	143		78	84	
Variants	Genotype									
C_1731723-10	A/A	171 (0.777)	173 (0.772)		114 (0.803)	108 (0.735)		57 (0.731)	65 (0.802)	
	A/T	45 (0.205)	50 (0.223)		25 (0.176)	35 (0.245)		20 (0.256)	15 (0.185)	
	T/T	4 (0.018)	1 (0.004)	0.3608	3 (0.021)	0 (0.000)	0.0896	1 (0.013)	1 (0.012)	0.5536
	Allele									
	A	387 (0.880)	396 (0.884)		253 (0.891)	251 (0.878)		134 (0.859)	145 (0.895)	
	T	53 (0.120)	52 (0.116)	0.8397	31 (0.109)	35 (0.122)	0.6218	22 (0.141)	17 (0.105)	0.3267
C_203475-1	T/T	173 (0.794)	179 (0.821)		113 (0.807)	115 (0.827)		60 (0.770)	64 (0.810)	
	T/C	42 (0.193)	37 (0.170)		24 (0.171)	22 (0.158)		18 (0.23)	15 (0.190)	
	C/C	3 (0.014)	2 (0.009)	0.7339	3 (0.021)	2 (0.014)	0.8603	0 (0.000)	0 (0.000)	N.C
	Allele									
	T	388 (0.890)	395 (0.906)		250 (0.893)	252 (0.906)		138 (0.885)	143 (0.905)	
	C	48 (0.110)	41 (0.094)	0.4336	30 (0.107)	26 (0.094)	0.5925	18 (0.115)	15 (0.095)	0.5547
C_1285859-10	G/G	3 (0.014)	3 (0.013)		3 (0.021)	2 (0.014)		0 (0.000)	1 (0.012)	
	G/A	42 (0.191)	36 (0.161)		24 (0.169)	22 (0.155)		18 (0.231)	14 (0.170)	
	A/A	175 (0.795)	185 (0.826)	0.7035	115 (0.810)	118 (0.831)	0.8498	60 (0.769)	67 (0.817)	0.4092
	Allele									
	G	48 (0.109)	42 (0.094)		30 (0.106)	26 (0.092)		18 (0.115)	16 (0.098)	
	A	392 (0.891)	406 (0.906)	0.4488	254 (0.894)	258 (0.908)	0.5734	138 (0.885)	148 (0.902)	0.605
C_1285838-1	C/C	48 (0.221)	46 (0.204)		36 (0.257)	27 (0.189)		12 (0.156)	19 (0.232)	
	C/T	85 (0.392)	112 (0.498)		55 (0.393)	74 (0.517)		30 (0.390)	38 (0.463)	
	T/T	84 (0.387)	67 (0.298)	0.0635	49 (0.350)	42 (0.294)	0.1007	35 (0.455)	26 (0.305)	0.133
	Allele									
	C	181 (0.417)	204 (0.453)		127 (0.454)	128 (0.448)		54 (0.351)	76 (0.463)	
	T	2353 (0.583)	246 (0.547)	0.2767	153 (0.546)	158 (0.552)	0.8856	100 (0.649)	88 (0.537)	0.0409

Significant difference in distribution; N.C.indicated the statistical result6 showing no calculating of chi-squre in a contingency table,due to including a cell with the 0 number of sample.

The haplotype-based case-control study also documented significant differences between the women of the EH and NT groups (Table 3). There were significant differences for the results of the A-T-A-C haplotype, with this haplotype higher in the EH (42.6%) than in the NT group (28.8%).

**Table 3:** Haplotype Distribution in NT controls EH patients

Haplotype	Total				Male				Female			
	NT	EH	Chi-Squ	*P value*	NT	EH	Chi-Squ	*P value*	NT	EH	Chi-Squ	*P value*

A-T-A-T	0.435	0.409	0.559	0.455	0.422	0.425	0.007	0.931	0.452	0.375	1.859	0.173
A-T-A-C	0.333	0.378	1.885	0.170	0.361	0.354	0.032	0.859	0.288	0.426	6.387	0.012
A-C-G-T	0.091	0.084	0.147	0.701	0.085	0.080	0.095	0.758	0.104	0.090	0.178	0.674
T-T-A-C	0.066	0.072	0.149	0.699	0.072	0.084	0.227	0.634	0.979	0.050	0.048	0.001
T-T-A-T	0.055	0.042	0.931	0.335	0.036	0.043	0.189	0.663	0.093	0.041	3.512	0.060
A-C-G-C	0.020	0.009	1.976	0.160	0.023	0.014	0.407	0.523	0.013	0.000	2.039	0.153
A-T-G-T	0.000	0.005	1.995	0.158	0.000	0.000	0.000	1.000	0.000	0.013	1.987	0.159
T-C-A-C	0.000	0.002	0.000	1.000	0.000	0.000	0.000	1.000	0.000	0.000	0.000	1.000
	X^2^=6.63, *P*=0.36				X^2^=0.90, *P*=0.97				X^2^=12.67, *P*=0.049			

Haplotype are shown as combined alleles of C_1731723-10,C_203475-10,C_125838-1.

## DISCUSSIONS

APA cleaves the N-terminal aspartyl acid residue of Ang II to produce Ang III. An accumulation of Ang II leads to an increase in blood pressure. A previous animal study that examined the APA deficient mouse documented a relationship between hypertension and the APA gene (1) That study indicated that the SBP and DBP of the APA-/- mouse were significantly higher than those seen in the APA+/- mouse and the APA+/+ mouse. Therefore, the metabolism of Ang II by APA is an important factor in the regulation of blood pressure (1-5, 18, 19). This suggests that the APA gene might be an indicator of disease susceptibility for EH. The use of SNPs is a valuable tool for association studies examining genomic markers. Since the SNPs consisted of 3 genotypes and 2 alleles, we were able to investigate the association between EH and the frequency of the genotype and the allele. We examined the association between the APA gene and EH using 4 SNPs of this gene. As there have been no previous association studies concerning the EH and APA gene, our study is the first one to attempt to determine this relationship.

In the present study, we found no differences in genotypic frequency between the EH and control groups. However, the allelic frequency of rs2290105 was significantly different between the women in the EH and NT groups. There have been previous reports on the APA knockout mouse, but these studies did not divide the mice into sexual groups (1). Therefore our data suggest that SNP rs2290105 might be a genetic marker for EH in women.

Genetic association studies have previously identified genes correlated with a gender-specific susceptibility to essential hypertension (20, 21). However, the reason for the present finding of a positive association between EH and SNP rs2290105 in women is unclear. There are other studies that have examined the association of the components of the renin-angiotensin system with gender-specific hypertension (22, 23). These observations highlight the potential importance of gender-dependent interactions between the genetic background and the expression of the hypertensive phenotype.

In conclusion, the present study is the first to examine the correlation between the human APA gene and EH. The present data indicate that the APA gene is a promising candidate for use as a genetic marker for EH in women.
